# Size in small-for-gestational-age neonates correlates with insulin-like growth factor–binding protein proteolysis and insulin-like growth factor-I bioavailability

**DOI:** 10.1210/jendso/bvag156

**Published:** 2026-07-06

**Authors:** Vicente Barrios, Álvaro Martín-Rivada, Gabriel Á Martos-Moreno, María Jiménez-Hernaiz, Ana Campillo-Calatayud, María Sánchez-Holgado, Adelina Pellicer, Julie A Chowen, Beatriz Corredor-Andrés, Leandro Soriano-Guillén, Jesús Argente

**Affiliations:** Department of Endocrinology, Hospital Infantil Universitario Niño Jesús, Research Institute “La Princesa,” Madrid E-28009, Spain; Centro de Investigación Biomédica en Red de Fisiopatología de la Obesidad y Nutriciόn (CIBEROBN), Instituto de Salud Carlos III, Madrid E-28009, Spain; Department of Endocrinology, Hospital Infantil Universitario Niño Jesús, Research Institute “La Princesa,” Madrid E-28009, Spain; Department of Endocrinology, Hospital Infantil Universitario Niño Jesús, Research Institute “La Princesa,” Madrid E-28009, Spain; Centro de Investigación Biomédica en Red de Fisiopatología de la Obesidad y Nutriciόn (CIBEROBN), Instituto de Salud Carlos III, Madrid E-28009, Spain; Department of Pediatrics, Universidad Autónoma de Madrid, Madrid E-28029, Spain; Department of Endocrinology, Hospital Infantil Universitario Niño Jesús, Research Institute “La Princesa,” Madrid E-28009, Spain; Department of Endocrinology, Hospital Infantil Universitario Niño Jesús, Research Institute “La Princesa,” Madrid E-28009, Spain; Department of Neonatology, Hospital Universitario La Paz, Hospital La Paz Institute for Health Research, IdiPAZ, Madrid E-28046, Spain; Department of Neonatology, Hospital Universitario La Paz, Hospital La Paz Institute for Health Research, IdiPAZ, Madrid E-28046, Spain; Department of Endocrinology, Hospital Infantil Universitario Niño Jesús, Research Institute “La Princesa,” Madrid E-28009, Spain; Centro de Investigación Biomédica en Red de Fisiopatología de la Obesidad y Nutriciόn (CIBEROBN), Instituto de Salud Carlos III, Madrid E-28009, Spain; IMDEA, CEIUAM+CSI, Food Institute, Cantoblanco, Madrid E-28049, Spain; Department of Endocrinology, Hospital Infantil Universitario Niño Jesús, Research Institute “La Princesa,” Madrid E-28009, Spain; Department of Pediatrics, Hospital Universitario Fundación Jiménez Díaz, Instituto de Investigación Fundación Jiménez Díaz, Madrid E-28040, Spain; Department of Endocrinology, Hospital Infantil Universitario Niño Jesús, Research Institute “La Princesa,” Madrid E-28009, Spain; Centro de Investigación Biomédica en Red de Fisiopatología de la Obesidad y Nutriciόn (CIBEROBN), Instituto de Salud Carlos III, Madrid E-28009, Spain; Department of Pediatrics, Universidad Autónoma de Madrid, Madrid E-28029, Spain; IMDEA, CEIUAM+CSI, Food Institute, Cantoblanco, Madrid E-28049, Spain

**Keywords:** IGF-I, IGFBP, neonate, pappalysin, ponderal index, small for gestational age, stanniocalcin

## Abstract

**Context:**

Alterations in prenatal growth affect postnatal outcomes, but the factors involved in prenatal growth are not clearly defined. Cord blood levels of insulin-like growth factor (IGF)-I are associated with weight and length at birth; however, how pappalysins (pregnancy-associated plasma protein [PAPP]-A and PAPP-A2) and stanniocalcins (STC-1 and STC-2) regulate IGF-binding protein (IGFBP) cleavage and IGF bioavailability in relation to birth size and nutritional status in full-term neonates is unknown.

**Objective:**

We aimed to determine the relationship between serum levels of PAPP-A and STCs with other components of the IGF axis and ponderal index (PI) in full-term small-for-gestational-age (SGA), adequate-for-gestational-age (AGA), or large-for-gestational-age (LGA) neonates (according to gestational age or PI).

**Methods:**

Twenty-six SGA, 131 AGA, and 22 LGA neonates were studied, and circulating levels of IGF axis parameters were evaluated according to weight, length, weight-to-length ratio, and PI.

**Results:**

Small-for-gestational-age newborns had lower total and free IGF-I, total IGFBP-3, acid-labile subunit, and PAPP-A2 levels and higher total IGFBP-1 and IGFBP-2, intact IGFBP-4, and STC-1 levels than AGA newborns. Neonates in the first tertile of PI had lower total and free IGF-I and IGFBP-5 and higher IGFBP-1 and -2 levels than those in the third tertile. Pregnancy-associated plasma protein-A2 levels correlated positively with weight and length and inversely with IGFBP-4 and STC-1 levels. Insulin-like growth factor-I and PAPP-A2 levels are associated with length.

**Conclusion:**

Variations in IGF-I parameters, such as STCs and PAPPAs, according to length/weight or PI suggest that they play an important role in intrauterine growth and/or nutritional status.

Fetal growth is determined by gestational age and genetic, environmental, and nutritional factors. Maternal and fetal genetic contributions account for approximately half of the variability in birth weight, with the remainder attributed to the intrauterine environment [1, 2]. The postnatal consequences of both low and high birth weights have received increasing attention in recent years, especially small-for-gestational-age (SGA) neonates whose weight and/or length are <−2 SD score (SDS) than that expected for their gestational age [3]. Between 2% and 2.5% of children are born SGA and, according to this classification used by pediatric endocrinologists, an equal proportion are large for gestational age (LGA). Small-for-gestational-age individuals are at risk of remaining small in adulthood, and both SGA and LGA neonates may develop metabolic alterations related to rapid postnatal weight gain [4, 5].

Insulin and insulin-like growth factors (IGFs) are important mitogens and determinants of fetal development [6]. It is well established that IGF-II is necessary for adequate growth and its overexpression is related to excessive fetal growth [7]; however, the actions of IGF-I on intrauterine growth and nutritional status are also increasingly recognized [8, 9]. The IGF-I levels in the cord blood of healthy neonates are directly correlated with birth weight and length [10, 11]. In transgenic mice, mutations in the *IGF-I* gene result in offspring that are 40% smaller than wild-type littermates, with adult size being even more affected [12]. Insulin-like growth factor-I is also considered to be indicative of nutrition during intrauterine life [13], with a direct relationship between IGF-I levels in cord blood and ponderal index (PI) [14].

Most circulating IGFs are bound to IGF-binding proteins (IGFBPs), which augments their half-life and modulates tissue distribution [15]. However, the role of IGFBP-3 in regulating fetal growth is unclear as levels of this binding protein, produced at the maternal–fetal interface, are reported to positively correlate with birth weight [16], but also to be higher in SGA neonates [17]. Moreover, mice overexpressing *Igfbp-3* have a lower birth weight than their littermate controls [18]. Binding of IGFs to IGFBPs also reduces their bioavailability and activity, whereas proteolytic cleavage of IGFBPs promotes IGF release and receptor activation [19, 20].

Pregnancy-associated plasma protein-A (PAPP-A) and PAPP-A2 are metalloproteinases that enhance IGF availability and actions [21-23], with their proteolytic activity being increased during gestation due to their synthesis in the placenta [24]. Pregnancy-associated plasma protein-A preferentially exerts its proteolytic actions on IGFBP-2, IGFBP-4, and IGFBP-5. Notably, IGFBP-4 must be bound to IGF-I or IGF-II for this pappalysin to exert its effects. Pregnancy-associated plasma protein-A2 cleaves IGFBP-3 and IGFBP-5 [24]. Stanniocalcin (STC)-1 and STC-2 are potent inhibitors of the proteolytic activity of PAPP-As and decrease IGF availability [25]. Transcriptome profiling of human placental gene expression showed that *STC-1* mRNA levels were highest in women who developed preeclampsia and delivered an SGA newborn, suggesting that STC-1 could be a prognostic biomarker during pregnancy [26].

Our understanding of the roles of PAPP-As and STCs in regulating IGF-I bioavailability during intrauterine growth, as well as their relationship with the nutritional status, is limited. Thus, the objectives of this study were to evaluate circulating levels of the IGF system parameters in full-term neonates according to both their size for gestational age and nutritional status, as determined by the PI. In addition, the correlation between these factors and birth weight, length, weight-to-length ratio (W/L), and PI at birth were analyzed.

## Materials and methods

### Ethical statement

The ethics committees of the Hospital Infantil Universitario Niño Jesús, Hospital Universitario Fundación Jiménez Díaz, and Hospital Universitario La Paz approved the study in accordance with the “Ethical Principles for Medical Research Involving Human Subjects” adopted in the Declaration of Helsinki by the World Medical Association. All parents were informed of the purpose of the study, and they provided their informed consent.

### Subjects

This cross-sectional, exploratory, and analytical study included a cohort of 179 healthy full-term neonates (90 males and 89 females). Neonates with a history of maternal pathology, including gestational diabetes, were excluded. Weight and length SDS were calculated according to national references [27]. Small-for-zgestational age subjects had a weight and/or length below −2 SDS, adequate for gestational age (AGA) between −2 and +2 SDS, and LGA greater than +2 SDS. To evaluate the intrauterine nutritional status, we calculated the W/L ratio as (weight [kg]/length [m]) and PI as (weight [g] × 100/length [cm]^3^). The subjects were classified into tertiles according to these indices. The weights, lengths, W/L ratios, and PI of the neonates are shown in Table 1.

**Table 1:** Anthropometric features of the full-term newborns included in the study

	Sex	SGA	AGA	LGA
Number	Male	14	64	12
	Female	12	67	10
GA (week)	Male	39.41 ± 0.96	39.34 ± 1.08	39.68 ± 1.31
	Female	39.00 ± 1.17	39.29 ± 0.94	39.49 ± 1.40
L (SDS)	Male	−2.17 ± 0.73	−0.28 ± 0.78*^e^*	1.52 ± 0.75*^c^*^,*e*^
	Female	−2.19 ± 0.82	−0.24 ± 0.78*^e^*	1.64 ± 1.04*^c^*^,*e*^
W (SDS)	Male	−1.84 ± 0.56	0.07 ± 0.95*^e^*	2.51 ± 0.65*^c^*^,*e*^
	Female	−2.03 ± 0.43	−0.02 ± 0.70*^e^*	2.24 ± 0.19*^c^*^,*e*^
W/L	Male	5.59 ± 0.54	6.76 ± 0.77*^e^*	8.32 ± 0.41*^c^*^,*e*^
	Female	5.13 ± 0.52*^d^*	6.43 ± 0.64^*d,e*^	7.89 ± 0.38*^c^*^,*d*,*e*^
PI (W/L^3^)	Male	2.56 ± 0.28	2.74 ± 0.28*^a^*	2.97 ± 0.23*^a,b^*
	Female	2.48 ± 0.24	2.68 ± 0.25*^a^*	2.88 ± 0.29*^a,b^*

Data are expressed as the mean ± SD.

Abbreviations: AGA, adequate (A) for gestational age (GA); L, length; LGA, large (L) for GA; PI, ponderal index; SDS, SD score (*Z*-score); SGA, small (S) for GA; W, weight; W/L, weight-to-length ratio.

^*a*^ *P* < .01, *P* < .001 of AGA and LGA vs SGA.

^*b*^ *P* < .01.

^*c*^ *P* < .001 of LGA vs AGA.

^*d*^ *P* < .05 of female vs male (intragroup).

^*e*^ *P* < .0001.

Cord blood was obtained at birth, and the samples were immediately centrifuged at 1800 *g* for 15 minutes at 4 °C. The serum was collected and maintained at −80 °C until biochemical measurements were performed.

### Biochemical determinations

Serum concentrations of total and free IGF-I, total IGF-II, total and intact IGFBP-3 and IGFBP-4, total IGFBP-5, PAPP-A, and STC-2 were obtained by performing enzyme-linked immunosorbent assays (ELISAs) (Ansh Labs, Webster, TX, USA). Serum acid-labile subunit (ALS; Mediagnost, Reutlingen, Germany), insulin (BioVendor, Brno, Czech Republic), and STC-1 levels were determined by using ELISAs (R&D Systems, Minneapolis, MN, USA). Pregnancy-associated plasma protein -A2 was measured with a chemiluminescent immunoassay (Cloud-Clone, Katy, TX, USA). The Research Resource Identifiers for the immunoassays employed in this study are listed in Table 2. All assays were performed according to the manufacturer's instructions, and the intra- and interassay coefficients of variation were <10% in all cases.

**Table 2:** Research Resource Identifier (RRID) of the assays used

Parameters	Commercial source	Catalog #	RRID
Total IGF-I	Ansh Labs	AL-121	AB_2783672
Free IGF-I	Ansh Labs	AL-122	AB_2783673
IGF-II	Ansh Labs	AL-131	AB_2783680
IGFBP-1	Mediagnost	E01	AB_2813788
IGFBP-2	Ansh Labs	AL-140	AB_2783686
Total IGFBP-3	Ansh Labs	AL-120	AB_2783671
Intact IGFBP-3	Ansh Labs	AL-149	AB_2783688
Total IGFBP-4	Ansh Labs	AL-126	AB_2783676
Intact IGFBP-4	Ansh Labs	AL-128	AB_2783678
IGFBP-5	Ansh Labs	AL-127	AB_2783677
ALS	Mediagnost	E35	AB_2813809
Insulin	BioVendor	RIS006R	AB_2893123
STC-1	R&D Systems	DY2958	AB_2893122
STC-2	Ansh Labs	AL-143	AB_2783687
PAPP-A	Ansh Labs	AL-101	AB_2783656
PAPP-A2	Cloud-Clone	SCD471Hu	AB_2893124

Abbreviations: ALS, acid-labile subunit; IGF, insulin-like growth factor; IGFBP, insulin-like growth factor–binding protein; PAPP, pregnancy-associated plasma protein; STC, stanniocalcin.

### Statistics

Demographic data are presented as mean ± SD. Serum concentrations of the components of the IGF system are reported as the median and interquartile range due to the nonnormality of variables. The Shapiro–Wilk test was used to analyze the normality of the variables. Differences in serum parameters were determined using the Kruskal–Wallis test, followed by pairwise Mann–Whitney *U* post hoc test with significance correction. The relationship between parameters was determined using Spearman's correlation. Multiple linear regression analysis with an explanatory purpose was performed to evaluate the association between anthropometric features and the IGF axis; additionally, the relationship between total IGF-I and the other components of the IGF axis was evaluated. Statistical data were analyzed by using Statview (Statview 5.01, SAS Institute, Cary, NC, USA) and GraphPad Prism (GraphPad Software 8, San Diego, CA, USA) software. Statistical significance was set at *P* < .05.

## Results

### The IGF-I system according to length and/or weight for gestational age

**Figure 1:**
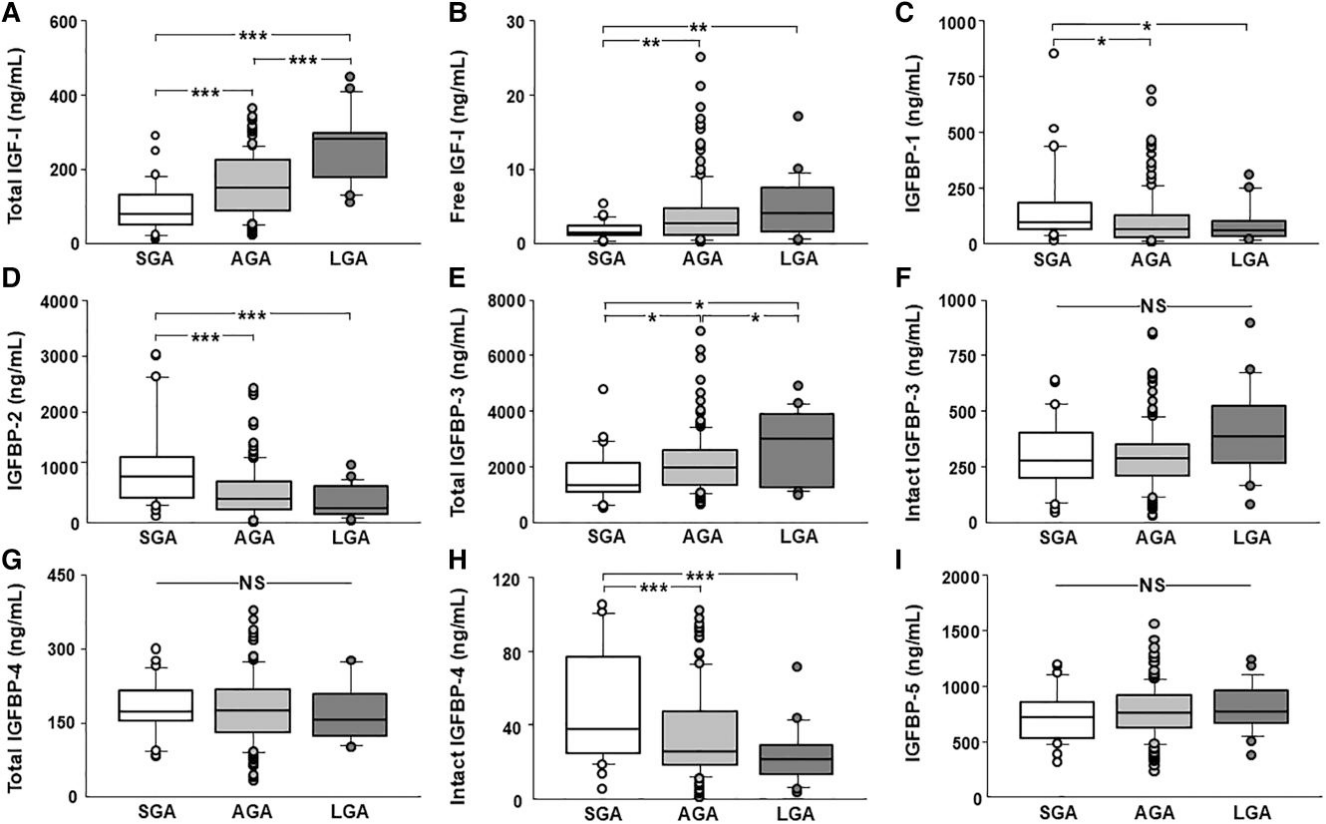
Box plots showing serum concentrations of total IGF-I (A), free IGF-I (B), IGFBP-1 (C), IGFBP-2 (D), total IGFBP-3 (E), intact IGFBP-3 (F), total IGFBP-4 (G), intact IGFBP-4 (H), and IGFBP-5 (I) in full-term neonates with small (S) weight and/or length for gestational age (GA), adequate for GA (AGA), or large for GA (LGA). The whiskers of the plots represent the 5 to 95 percentiles. The lower, middle, and upper lines of the box represent the lower, median, and upper quartiles, respectively. The points represent outliers. NS, nonsignificant. *P < .05, **P < .01, ***P < .001 by Kruskal–Wallis test followed by Mann–Whitney *U* post hoc test with significance correction.

No intragroup differences between the sexes were observed. Levels of total and free IGF-I and ALS were reduced in SGA compared to AGA and LGA, with an increase in total IGF-I and ALS in LGA compared to AGA (Fig. 1A and 1B, respectively). There were no differences in IGF-II levels. Insulin-like growth factor–binding protein-1 and IGFBP-2 levels were increased in SGA compared to AGA and LGA neonates, with no differences between the latter 2 groups (Fig. 1C and 1D, respectively).

Total IGFBP-3 levels were decreased in SGA and increased in LGA neonates compared to AGA (Fig. 1E), with no differences between groups in intact IGFBP-3 and total IGFBP-4 levels (Fig. 1F and 1G, respectively). Intact IGFBP-4 levels were higher in SGA than in AGA and LGA neonates (Fig. 1H). When these changes were determined in molar concentrations, the increases in total and intact IGFBP-3 between LGA and SGA neonates were 57.83 and 4.10 nmol/L, respectively. For the total and intact fractions of IGFBP-4, the changes were −0.42 and −0.46 nmol/L.

**Figure 2:**
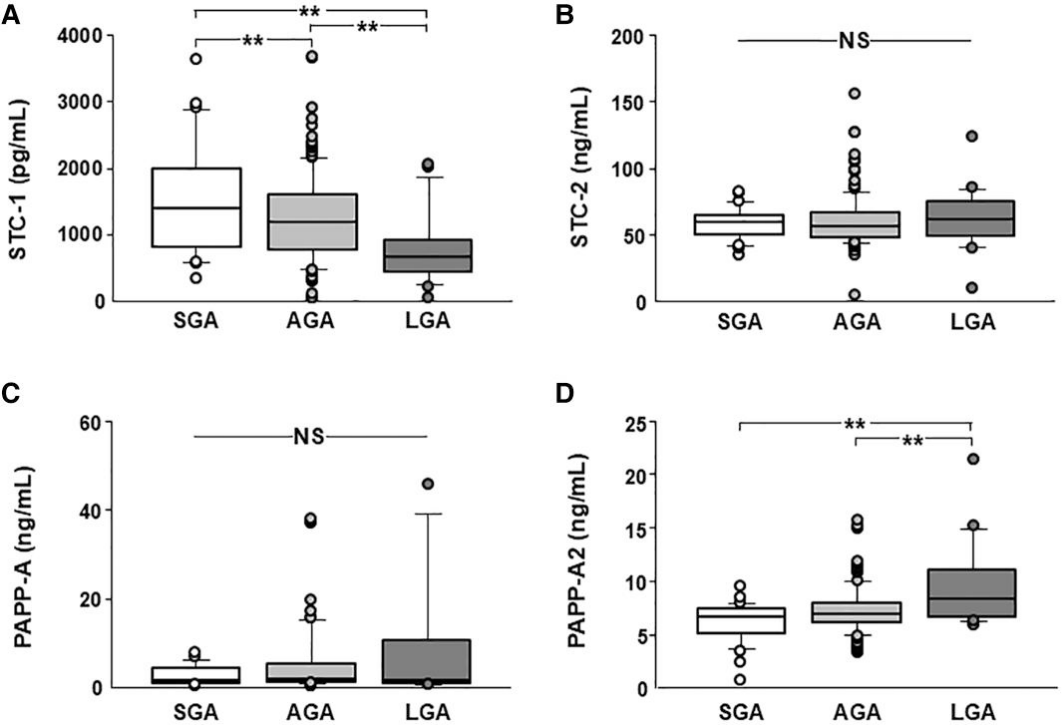
Box plots showing serum concentrations of STC-1 (A), STC-2 (B), PAPP-A (C), and PAPP-A2 (D) in full-term neonates with small (S) weight and/or length for gestational age (GA), adequate for GA (AGA), or large for GA (LGA). The whiskers of the plots represent the 5 to 95 percentiles. The lower, middle, and upper lines of the box represent the lower, median, and upper quartiles. The points represent outliers. NS, nonsignificant. **P < .01 by Kruskal–Wallis test followed by Mann–Whitney *U* post hoc test with significance correction.

Insulin-like growth factor–binding protein-5 levels did not differ between the groups (Fig. 1I), and insulin was higher in LGA. STC-1 levels were higher in SGA and lower in LGA neonates compared to those that were AGA (Fig. 2A), with no differences in STC-2 (Fig. 2B). Serum PAPP-A levels did not differ between groups (Fig. 2C), and PAPP-A2 levels were higher in LGA neonates than in those that were SGA or AGA (Fig. 2D).

### Changes in the IGF-I system according to weight/length ratio and PI

The median and interquartile ranges for each parameter according to the W/L ratio are presented in Table 3. Total and free IGF-I were higher in tertiles 2 and 3 than in tertile 1, with higher total IGF-I in tertile 3 compared to those in tertile 2. No differences were observed in IGF-II concentrations. Insulin-like growth factor–binding protein-1 and IGFBP-2 levels were higher in tertiles 2 and 3. Levels of intact IGFBP-3, IGFBP-5, and ALS were higher, while levels of intact IGFBP-4 and STC-1 were lower in tertile 3 compared to those in tertiles 1 and 2.

**Table 3:** Weight-to-length ratio values and serum levels of the insulin-like growth factor system components in full-term newborns according to the division of weight-to-length ratio in tertiles

	Tertile 1	Tertile 2	Tertile 3
Weight/length	0.55 ± 0.05	0.66 ± 0.02*^c^*	0.78 ± 0.05*^c,d^*
T IGF-I, ng/mL	96.9 (58.6-138.7)	150.2 (89.5-227.1)*^c^*	209.3 (140.2-283.9)*^c,d^*
F IGF-I, ng/mL	1.54 (0.86-3.10)	2.80 (1.18-6.47)*^b^*	3.57 (1.37-5.74)*^b^*
IGF-II, ng/mL	105.8 (71.8-125.7)	103.8 (77.0-134.9)	103.1 (88.8-131.5)
IGFBP-1, ng/mL	91.1 (57.1-176.9)	55.9 (21.0-119.5)*^b^*	52.2 (21.9-114.0)*^b^*
IGFBP-2, ng/mL	626 (371-958)	363 (137-731)*^c^*	291 (169-579)*^c^*
T IGFBP-3, ng/mL	1995 (1483-2590)	1932 (1254-2812)	1988 (1285-3159)
I IGFBP-3, ng/mL	274 (207-343)	276 (205-368)	335 (240-426)*^a,e^*
T IGFBP-4, ng/mL	185.6 (143.7-218.7)	184.5 (155.1-226.7)	171.9 (118.4-213.2)
I IGFBP-4, ng/mL	24.6 (16.8-43.8)	24.5 (18.1-54.6)	19.1 (15.0-39.8)*^a,e^*
IGFBP-5, ng/mL	750 (682-893)	784 (622-915)	792 (696-972)*^a^*
Insulin, µU/mL	2.30 (0.98-3.75)	2.35 (1.26-4.73)	2.32 (1.61-6.77)
ALS, ng/mL	2157 (1889-2420)	2185 (1880-2456)	2349 (2071-2905)*^b,e^*
STC-1, pg/mL	1201 (684-1704)	1130 (589-1684)	891 (633-1361)*^a,e^*
STC-2, ng/mL	56.4 (50.8-67.5)	56.0 (48.2-65.8)	57.4 (46.6-66.4)
PAPP-A, ng/mL	1.83 (1.05-6.78)	2.00 (1.18-5.90)	1.94 (1.19-5.03)
PAPP-A2, ng/mL	6.92 (6.28-7.89)	6.80 (6.18-7.68)	6.75 (6.10-8.28)

Weight/length is expressed as the mean ± SD. Biochemical values are expressed as medians and interquartile ranges.

Abbreviations: ALS, acid-labile subunit; F, free; I, intact; IGF, insulin-like growth factor; IGFBP, insulin-like growth factor–binding protein; PAPP, pregnancy-associated plasma protein; STC, stanniocalcin; T, total.

^*a*^ *P* < .05.

^*b*^ *P* < .01.

^*c*^ *P* < .001 for tertile 2 and 3 vs tertile 1.

^*d*^ *P* < .001 for tertile 3 vs tertile 2.

^*e*^ *P* < .001 for tertile 3 vs tertiles 1 and 2.

**Figure 3:**
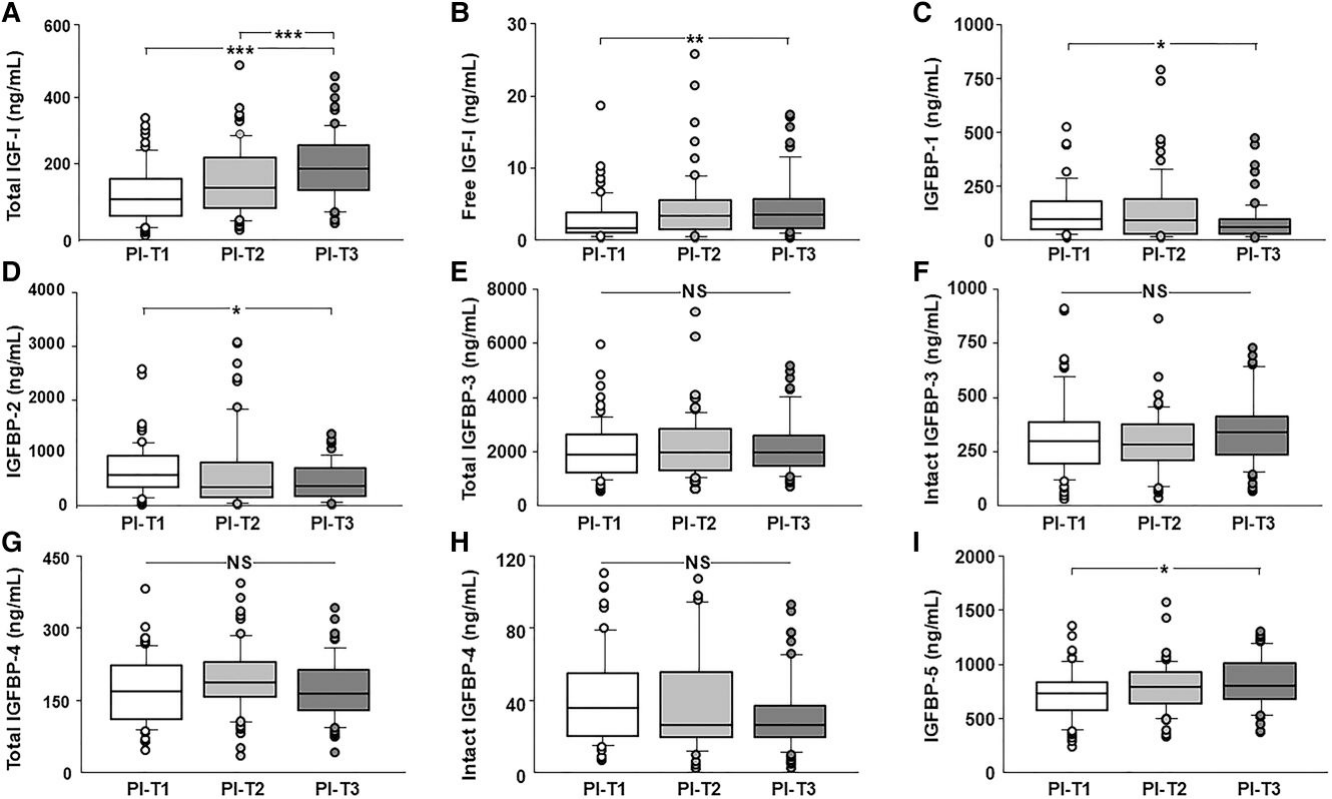
Box plots showing serum concentrations of total IGF-I (A), free IGF-I (B), IGFBP-1 (C), IGFBP-2 (D), total IGFBP-3 (E), intact IGFBP-3 (F), total IGFBP-4 (G), intact IGFBP-4 (H), and IGFBP-5 (I) in full-term neonates according to ponderal index (PI) tertiles (T). The whiskers of the plots represent the 5 to 95 percentiles. The lower, middle, and upper lines of the box represent the lower, median, and upper quartiles. The points represent outliers. NS, nonsignificant. *P < .05, **P < .01, ***P < .001 by Kruskal–Wallis test followed by Mann–Whitney *U* post hoc test with significance correction.

According to PI, total IGF-I levels were lower in neonates in tertiles 1 and 2 compared to those in tertile 3 (Fig. 3A), and free IGF-I levels were lower in tertile 1 compared to tertile 3 (Fig. 3B). Insulin-like growth factor–binding protein-1 and IGFBP-2 concentrations were higher in tertile 1 than in tertile 3 (Fig. 3C and 3D, respectively), with no differences in total or intact IGFBP-3 and IGFBP-4 levels (Fig. 3E, 3F, 3G, and 3H, respectively). Circulating IGFBP-5 was lower in tertile 1 than in tertile 3 (Fig. 3I). No differences in STC-1, STC-2, PAPP-A, or PAPP-A2 levels or the IGF and IGFBP ratios were observed among the groups (data not shown).

### Correlations and multiple regression analyses

The relationships between anthropometric data and biochemical parameters are shown in Table 4. Length and weight SDS values, as well as W/L and PI indexes, were directly correlated with total and free IGF-I and ALS concentrations and inversely with IGFBP-2. Weight-to-length ratio and PI positively correlated with IGFBP-5 levels. Length and weight SDS, but not PI values, were directly correlated with total IGFBP-3 and inversely with intact IGFBP-4 and STC-1 concentrations. The remaining significant correlations observed are presented in Table 4.

**Table 4:** Correlation between length, weight, weight for length ratio, and ponderal index with parameters of the IGF system in full-term newborns

	Length (SDS)		Weight (SDS)		W/L		PI (W/L^3^)	
	*r*_s_	*P*	*r*_s_	*P*	*r*_s_	*P*	*r*_s_	*P*
T IGF-I	**0**.**39**	**<**.**001**	**0**.**55**	**<**.**001**	**0**.**49**	**<**.**001**	**0**.**38**	**<**.**001**
F IGF-I	**0.32**	**<.001**	**0.38**	**<.001**	**0.30**	**<.001**	**0.23**	**<.01**
IGF-II	−0.02	.73	0.07	.36	0.08	.32	0.12	.09
IGFBP-1	−0.12	.12	**−0.28**	**<.001**	**−0.25**	**<.01**	**−0.23**	**<.01**
IGFBP-2	**−0.35**	**<.001**	**−0.39**	**<.001**	**−0.33**	**<.001**	**−0.19**	**<.05**
T IGFBP-3	**0.24**	**<.01**	**0.20**	**<.05**	**0.18**	**<.05**	0.05	.53
I IGFBP-3	0.07	.38	0.14	.07	0.15	.05	0.14	.05
T IGFBP-4	−0.06	.40	−0.01	.88	−0.07	.34	−0.02	.78
I IGFBP-4	**− 0.27**	**<.001**	**− 0.30**	**<.001**	**0.27**	**<.01**	− 0.12	.10
IGFBP-5	0.01	.95	0.14	.05	**0.24**	**<.01**	**0.26**	**<.01**
Insulin	0.15	.05	**0.21**	**<.01**	**0.20**	**<.05**	0.13	.09
ALS	**0.29**	**<.001**	**0.38**	**<.001**	**0.33**	**<.001**	**0.26**	**<.01**
STC-1	**−0.28**	**<.001**	**−0.22**	**<.01**	**−0.23**	**<.01**	−0.03	.67
STC-2	−0.03	.73	−0.03	.66	−0.01	.92	−0.05	.46
PAPP-A	0.07	.39	0.02	.72	0.03	.74	0.02	.81
PAPP-A2	**0.32**	**<.001**	0.06	.41	0.04	.63	0.09	.23

Correlations were evaluated using Spearman's correlation. Bold indicates statistically significant values.

Abbreviations: ALS, acid-labile subunit; F, free; I, intact; IGF, insulin-like growth factor; IGFBP, insulin-like growth factor–binding protein; L, length; PAPP, pregnancy-associated plasma protein; PI, ponderal index; STC, stanniocalcin; T, total; W, weight.

Significant associations between anthropometric data and parameters of the IGF-I system are shown in Table 5. IGF-I levels were positively associated with all indices. Insulin-like growth factor-II, insulin, and ALS were positively associated with length and weight, and PAPP-A2 with length, while IGFBP-2 was negatively associated with length.

**Table 5:** Multiple regression analyses for length, weight, weight for length ratio, and ponderal index with parameters of the IGF system in full-term newborns

Length (SDS)	Coefficient	*P*	95% CI	Model *R*^2^
T IGF-I	0.0028	<.01	0.0005 to 0.0051	0.33
IGF-II	0.0042	<.05	0.0009 to 0.0075	
IGFBP-2	−0.0004	<.05	−0.0007 to −0.0001	
Insulin	0.0259	<.0001	0.0128 to 0.0389	
ALS	0.0003	<.05	0.0001 to 0.0005	
PAPP-A2	0.1004	<.01	0.0354 to 0.1654	
**Weight (SDS)**				
T IGF-I	0.0057	<.0001	0.0035 to 0.0079	0.40
IGF-II	0.0029	<.05	0.0004 to 0.0062	
Insulin	0.0273	<.001	0.0130 to 0.0416	
ALS	0.0003	<.05	0.0001 to 0.0005	
**W/L**				
IGF-I	0.0004	<.0001	0.0002 to 0.0006	0.26
**PI (W/L^3^)**				
T IGF-I	0.0011	<.001	0.0005 to 0.0018	0.19

Abbreviations: ALS, acid-labile subunit; F, free; I, intact; IGF, insulin-like growth factor; IGFBP, insulin-like growth factor–binding protein; L, length; PAPP, pregnancy-associated plasma protein; PI, ponderal index; SDS, SD score (Z-score); T, total; W/L, weight-to-length ratio.

The associations between IGF-I and the other components of the IGF-I system are shown in Table 6. IGFBP-2 and STC-1 negatively correlated with IGF-I and free IGF-I concentrations, while intact IGFBP-3, IGFBP-5, ALS, and PAPP-A2 positively correlated with IGF-I levels.

**Table 6:** Multiple regression analyses of serum insulin-like growth factor-I levels in the IGF system in full-term newborns

IGF-I	Coefficient	*P*	95% CI	Model *R*^2^
FIGF-1	4.800	<.0001	2.397 to 7.202	0.61
IGFBP-2	−0.068	<.0001	−0.090 to −0.047	
I IGFBP-3	0.137	<.0001	0.074 to 0.199	
IGFBP-5	0.071	<.01	0.023 to 0.118	
ALS	0.019	<.01	0.005 to 0.033	
STC-1	−0.022	<.01	−0.036 to −0.007	
PAPP-A2	4.349	<.05	0.388 to 8.309	

Abbreviations: ALS, acid-labile subunit; F, free; FIGF, freeIGF1; I, intact; IGF, insulin-like growth factor; IGFBP, IGF-binding protein; L, length; PAPP, pregnancy-associated plasma protein; STC, stanniocalcin.

## Discussion

Our study indicates that most components of the IGF axis are modified in SGA neonates, with decreased levels of IGF-I, IGFBP-3, and ALS, together with increased levels of IGFBP-1 and IGFBP-2. Lower levels of IGF-I could result in a reduction in its formation of complexes with IGFBP-4, leading to reduced PAPP-A-mediated proteolysis of this binding protein and consequently higher levels of intact IGFBP-4, despite normal serum PAPP-A levels [28]. In addition, elevated STC-1 concentrations could inhibit PAPP-A activity, contributing to the accumulation of intact IGFBP-4, as demonstrated in normal-sized neonates with isolated congenital growth hormone (GH) deficiency [29]. Nevertheless, differences in STC-1, PAPP-A2 and intact IGFBP-4 were not observed when neonates were grouped according to their PI, with the changes in serum-free IGF-I levels being less pronounced. Lower IGFBP-5 levels in neonates with a reduced PI and the positive correlation between these factors suggest that IGFBP-5 could be a useful marker of intrauterine nutritional status.

A common feature in SGA is reduced levels of ternary complex components [30], and these factors are increased in LGA neonates [31], which could be an indication of anabolic processes. The increased IGFBP-1 and IGFBP-2 levels observed in SGA could reduce IGF-I bioavailability and have been associated with malnutrition [32]. Lower levels of IGF-I and ALS, as well as slightly lower levels of intact IGFBP-3 in SGA, could be interpreted in the framework of fetal programming and the thrifty phenotype hypothesis. Indeed, GH resistance is suggested to be a metabolic adaptation to early life nutritional deprivation, particularly in SGA [33].

The W/L ratio and PI are asymmetrical indices that provide an estimation of nutritional status [34, 35]. Differences in total and free IGF-I, ALS, and IGFBP-1 and IGFBP-2 values were less remarkable when using these indices than when stratified according to length or weight. Although previous studies reporting changes in these parameters according to PI suggest that they are nutritional markers [36, 37], our findings indicate that they are more closely related to length and weight. However, changes in IGFBP-5 levels according to W/L and PI support its possible relationship with the intrauterine nutritional status. Time-related changes in uterine IGFBP-5 expression during pregnancy are reported to play a role in determining placental size in relation to nutritional status [38].

Two of the most relevant findings reported here are the higher levels of intact IGFBP-4 and the lower concentrations of free IGF-I in SGA neonates. Intact IGFBP-4 is negatively associated with birth weight and length [39]. Although the higher levels of free IGF-I observed in AGA and LGA neonates are related to total IGF-I concentrations, it is also possible that increased proteolysis of IGFBP-4 releases IGF-I, thereby contributing to higher circulating free IGF-I levels in these neonates. We found lower levels of intact IGFBP-4 and higher levels of free IGF-I in neonates classified in the third tertile according to their W/L ratio. Higher free IGF-I levels were also observed in neonates in the third tertile according to PI, whereas those with lower W/L ratio or PI appear to exhibit reduced IGF-I bioavailability. This restriction could be driven by a change in binding proteins, where intact IGFBP-4 remains uncleaved or accumulates due to dysregulation of the PAPP-A pathway, thereby limiting the availability of free IGF-I to promote tissue growth [40].

Pregnancy-associated plasma protein-A cleaves IGFBP-4 into fragments, prompting the release of IGF-I to subsequently bind and stimulate its receptor [41]. Although no differences in PAPP-A levels were detected, increased STC-1 levels in SGA neonates suggest a potential reduction in PAPP-A activity, given that STC-1 acts as a competitive PAPP-A inhibitor [42]. Consequently, elevated STC-1 could reduce PAPP-A activity, thereby reducing the release of IGF-I from IGFBP-4–IGF-I complexes and leading to lower free IGF-I availability in SGA. Indeed, during embryogenesis, STC-1 is expressed in the appendicular skeleton and directly inhibits longitudinal bone growth at the growth plate [43]. Conversely, the decrease in STC-1 observed in neonates within the highest tertile of the W/L index is noteworthy, as STC-1 levels tend to decline in LGA neonates [26]. Finally, dysregulation of STC-1 has been linked to impaired trophoblast invasion and poor placental perfusion during early pregnancy [44] which could affect fetal growth. Changes in free IGF-I according to PI parallel the increase in total IGF-I concentrations, which could indicate that similar percentages of IGF-1 are released as no changes in PAPP-As or STCs were found. However, other proteinases, such as different a disintegrin and metalloproteinase enzymes could be involved in this process [45].

The levels of intact IGFBP-3 were unchanged despite the progressive increase in the total levels in direct relationship with weight and/or length. This suggests that there is more proteolysis of IGFBP-3 in LGA to maintain high levels of free IGF-I in these neonates [46], allowing its intrauterine growth-promoting actions [47]. This could be related to the increase in PAPP-A2 levels, which could trigger IGFBP-3 proteolysis [48] and favor IGF-I bioavailability. Pregnancy-associated plasma protein-A2 is expressed at the feto-maternal interface of the placenta throughout gestation and in embryos and modulates cartilage development [49]. There was a direct relationship between PAPP-A2 levels in cord blood and weight and length, which is in agreement with these growth-promoting actions.

It is important to note that relative changes in circulating IGFBPs do not reflect the overall impact that they may have on circulating IGF levels. For example, the molar concentrations of both total and intact IGFBP-3 are substantially higher than those of IGFBP-4. Thus, relative increases in the fractions of IGFBP-3 could have a more substantial effect than similar decreases in total and intact IGFBP-4. Although the intact fraction of IGFBP-3 increases, this rise is less than expected relative to the rise in its total levels. Consequently, while this shift may partially alter the bioavailability of IGF-I [50], it also secures a stable, continuous hormonal reservoir of IGF-I, prolongs its half-life, and protects it against protease degradation [51].

Insulin-like growth factor-I levels showed the strongest correlation with all anthropometric parameters in the correlation analyses, and association studies also indicated a positive relationship between this growth factor and anthropometric indices. It is suggested that IGF-I exerts more potent effects than IGF-II on growth at the end of gestation [52], and the association between PAPP-A2 and birth length observed corroborates the suggestion that this pappalysin favors IGF-I bioavailability and growth during the third trimester of gestation [53]. There is an inverse association between IGF-I and STC-1, with STC1 proposed to act as a negative regulator of fetal growth [43].

We did not analyze the possible effect of proteolysis of IGFBP-1, IGFBP-2, and IGFBP-5 on IGF-I bioavailability as reliable methods for the determination of intact fractions of these binding proteins are not available. However, IGFBP-1 and IGFBP-2 are present at high concentrations in the cord blood [14, 20] and are known to inhibit IGF-I actions [54]. Likewise, analyzing the activities of PAPP-As and STCs would provide a more in-depth understanding of the physiology of the IGF axis at birth. Indeed, although associations have been established among several parameters of this axis, these findings should be interpreted with caution as the interactions between these factors are most likely more complex, and they should also take into consideration other factors such as time and tissue-specific changes that cannot be analyzed employing cord blood samples.

In conclusion, this study furthers our understanding of the regulation of the peripheral GH-IGF axis in full-term neonates and shows that intrauterine growth and/or nutritional status could be related to differences in various IGF-I parameters, as these change according to length, weight, and PI. Indeed, both intrauterine growth and nutritional status were found to be associated with levels of stanniocalcins and pappalysins, factors that control the bioavailability of IGFs by modulating the proteolysis of IGFBPs. Further studies are needed to better understand the role of the various components of the IGF-I system in fetal and neonatal growth and how they can modulate nutritional status during intrauterine life.

## Data Availability

Some or all datasets generated during and/or analyzed during the present study are not publicly available but are available from the corresponding author upon reasonable request.

## Abbreviations

AGA: adequate for gestational age
ALS: acid-labile subunit
ELISA: enzyme-linked immunosorbent assay
IGF: insulin-like growth factor
IGFBP: IGF-binding protein
LGA: large for gestational age
PAPP: pregnancy-associated plasma protein
PI: ponderal index
SDS: SD score
SGA: small for gestational age
STC: stanniocalcin
W/L: weight-to-length ratio
